# RACIAL BIAS IN STAFF OPTIMISM ABOUT ADL IMPROVEMENT AMONG NURSING HOME RESIDENTS

**DOI:** 10.1093/geroni/igz038.928

**Published:** 2019-11-08

**Authors:** Kenneth J Branco

**Affiliations:** Stonehill College, Easton, United States

## Abstract

Research on bias in health care has shown disparity in provision of care to and health outcomes of African Americans. Patient optimism was associated with improved physical and psychosocial outcomes, and nurse optimism was correlated with patient perceptions of care. We estimated effects of race using logistic regression, controlling for ADLs, cognitive impairment, and gender on both staff optimism and resident optimism about capacity for improvement in ADLs in a probability sample (n=2604) of nursing home residents who were evaluated with the resident assessment instrument (RAI). We found no difference between African American and White residents’ optimism about their own capacity for improvement. Staff findings were quite different. Staff were most optimistic about the potential of residents who needed ADL assistance OR=1.82; 95% CI [1.42-2.32] over those who were ADL dependent or those who only needed oversight. Most importantly, it was in the oversight category of ADL impairment where the greatest indication of racial prejudice occurred. Staff were much less likely to be optimistic about African American residents (16%) compared to White residents (30%). With all control variables entered, staff were still less willing to be optimistic about African American resident improvement (AOR=0.65; 95% CI [0.44-0.96]. The implications of these findings are troubling. It is unlikely that staff would expend energy on improving the functioning of the African American residents whom they believe cannot improve. Further research is needed on the extent to which prejudice in nursing homes is accompanied by discrimination and how the bias can be overcome.

